# Resolving the Detailed Structure of Cortical and Thalamic Neurons in the Adult Rat Brain with Refined Biotinylated Dextran Amine Labeling

**DOI:** 10.1371/journal.pone.0045886

**Published:** 2012-11-05

**Authors:** Changying Ling, Michael L. Hendrickson, Ronald E. Kalil

**Affiliations:** 1 Department of Surgery, School of Medicine and Public Health, University of Wisconsin-Madison, Madison, Wisconsin, United States of America; 2 W.M. Keck Laboratory for Biological Imaging, School of Medicine and Public Health, University of Wisconsin-Madison, Madison, Wisconsin, United States of America; 3 Department of Ophthalmology and Visual Sciences, School of Medicine and Public Health, University of Wisconsin-Madison, Madison, Wisconsin, United States of America; University of Edinburgh, United Kingdom

## Abstract

Biotinylated dextran amine (BDA) has been used frequently for both anterograde and retrograde pathway tracing in the central nervous system. Typically, BDA labels axons and cell somas in sufficient detail to identify their topographical location accurately. However, BDA labeling often has proved to be inadequate to resolve the fine structural details of axon arbors or the dendrites of neurons at a distance from the site of BDA injection. To overcome this limitation, we varied several experimental parameters associated with the BDA labeling of neurons in the adult rat brain in order to improve the sensitivity of the method. Specifically, we compared the effect on labeling sensitivity of: (a) using 3,000 or 10,000 MW BDA; (b) injecting different volumes of BDA; (c) co-injecting BDA with NMDA; and (d) employing various post-injection survival times. Following the extracellular injection of BDA into the visual cortex, labeled cells and axons were observed in both cortical and thalamic areas of all animals studied. However, the detailed morphology of axon arbors and distal dendrites was evident only under optimal conditions for BDA labeling that take into account the: molecular weight of the BDA used, concentration and volume of BDA injected, post-injection survival time, and toning of the resolved BDA with gold and silver. In these instances, anterogradely labeled axons and retrogradely labeled dendrites were resolved in fine detail, approximating that which can be achieved with intracellularly injected compounds such as biocytin or fluorescent dyes.

## Introduction

Experimental methods have been used to study neurons and their connectivity in the central nervous system (CNS) for many decades [1]. In 1908, Bielschowsky [2] introduced a silver staining method for axons and neurofibrils that is still used today by neuropathologists [3]. Nearly 60 years ago, Nauta and Gygax [4] modified Bielschowsky's method to demonstrate that metallic silver could be used to impregnate degenerating axons selectively. Several years later, Fink and Heimer [5] refined this selective silver staining method to resolve the terminal boutons of degenerating axons. Subsequently, however, the selective staining of degenerating axons as a method for studying connectivity in the CNS was all but replaced by methods that exploited the capability of neurons to transport various biomolecules anterogradely and retrogradely.

Among the many transported molecules that have been used to trace neural connections are radioactive amino acids [6], horseradish peroxidase [7], conjugates of horseradish peroxidase (HRP) such as wheat germ agglutinin-HRP [8]–[10], biocytin [11], [12], cholera toxin [13]–[15], and *Phaseolus vulgaris* leucoagglutinin [16]. While these various methods, and others not mentioned here, have provided important insights into fundamental aspects of neuronal connectivity, they have offered relatively limited information about the detailed structure of the dendritic and axonal arbors of the cells involved. However, resolving the morphology and spatial distribution of the dendritic and axonal arbors of neurons has been a topic of interest for many years in the belief that knowledge of neuron structure is important in understanding neuron function.

To reveal information about neuronal structure, various silver staining methods and numerous modifications have been developed that all trace their origin to the “dark reaction” introduced by Golgi in 1875 [17], [18]. However, while different Golgi methods are relatively easy to carry out and often have yielded excellent results, they are not without shortcomings. Foremost among these limitations is the frequently unpredictable and quixotic staining of neurons when Golgi methods are applied. Many tracer molecules, such as HRP and its various conjugated forms, can be injected extracellularly into the CNS, and will be taken up by cells in the vicinity of the injection site, but when delivered extracellularly these molecules mainly label nerve cell somas and proximal neurites. While *Phaseolus vulgaris* leucoagglutinin has been used to label distal neuronal processes, transport occurs only anterogradely and the efficiency of labeling is low if pressure rather than iontophoretic injections are employed [16].

By contrast, the intracellular injection of water soluble, aldehyde-fixable fluorescent tracers such as Lucifer Yellow CH or Cascade Blue or molecules such as biocytin or HRP that can be resolved chrogenically, are capable of cell labeling that reveals the complete structure of the injected cell [19]. However, the intracellular injection of single cells is technically challenging, especially *in vivo*, and is not well suited to studying large populations of neurons in the mammalian brain. Thus, despite the variety of available methods for studying the morphology of neurons in the CNS, there are few that are both reliable and sensitive, can be applied *in vivo*, and are capable of labeling individual neurons in detail without requiring single cell injections.

Biotinylated dextran amine (BDA) has many advantages for labeling neurons and tracing neuronal pathways. After being injected into the CNS, BDA can be transported rapidly from the injection site both anterogradely and retrogradely [1], can be visualized using uncomplicated procedures, and sometimes can yield neuronal labeling that is Golgi-like in its detail. However, BDA-labeling of neurons often is incomplete. Proximal processes of labeled neurons typically are rendered satisfactorily, but fine distal elements often are incompletely filled and poorly resolved [20]–[29].

To determine if BDA can be used reliably for detailed neuronal labeling, we varied several experimental conditions that have been reported to affect BDA labeling, using projection neurons in the rat dorsal lateral geniculate nucleus (dLGN) and neurons in the visual cortex to evaluate the effects of modifying specific parameters thought to influence BDA labeling of neurons in the CNS. We chose these two sites because the morphology of neurons in the rat dLGN and visual cortex and their connections have been studied extensively with a variety of techniques [30]–[47], and the information that has been compiled provides an extensive, normative database for evaluating the effectiveness of different BDA labeling conditions. On balance, the labeling results reported here indicate that BDA is well-suited to reveal fine neuronal morphology when it is administered and subsequently resolved under optimal conditions.

## Materials and Methods

### Ethics Statement

#### Research Animals

All animal handling and procedures were performed in accordance with protocols for these studies that have been approved by the Institutional Animal Care and Use Committee at the University of Wisconsin-Madison. All surgery was performed aseptically under deep anesthesia and every attempt was made to minimize pain and discomfort.

### Research Animals

#### Labeling of dLGN Projection Neurons with BDA

**Figure 1:**
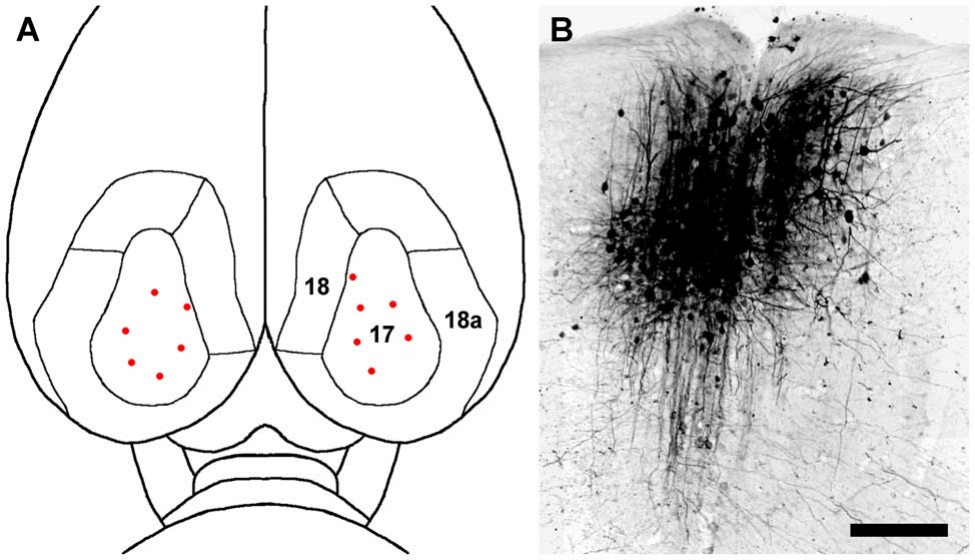
Typical bilateral BDA injection sites in the visual cortex. (**A**) A dorsal view of the cortical visual areas of the rat brain, indicated by the numbers 17, 18 and 18a [36]–[38], is shown. The locations of multiple BDA injection sites are represented by red dots. (**B**) A photomicrograph in the coronal plane showing a typical site for a 0.1 µL injection of 3,000 MW BDA in cortical area 17. Scale bar: 3.0 mm in **A**, 125 µm in **B**.

Thirty-one adult male Holtzman rats (250–275 g) were used in this study. Animals were anesthetized initially with an intramuscular injection of a mixture of 90 mg/kg of ketamine HCl and 9 mg/kg of xylazine. The animals then were placed in a stereotaxic head holder, and anesthesia during surgery was maintained with 1–2% isoflurane. Under aseptic conditions, bone flaps were cut to expose the dorsal surface of the visual cortex bilaterally as previously defined [36]–[38]. A 5% w/v solution of BDA (3,000 MW or 10,000 MW, Invitrogen) in double distilled water, or a 1∶1 mixture by volume of 5% w/v BDA and NMDA (10 mM or 100 mM, Sigma), in phosphate buffered saline, was pressure-injected into the visual cortex through a glass pipette with a tip diameter of approximately 10 µm. The injections largely were restricted to cortical area 17, but one or two of them may have encroached upon the border between area 17 and area 18 (Fig. 1A). However, since area 18 does not appear to receive a projection of any substance from the dLGN [36], it is unlikely that the possible encroachment had any effect on the retrograde labeling of dLGN projection neurons.

The tip of the injection pipette was lowered to approximately 0.8 mm below the cortical surface of the brain to center the injection in layer 4 of the cortex (Fig. 1B). The volume of each injection of BDA by itself was 0.02–0.4 µl; the volume of the injections that combined BDA with NMDA was 0.1–0.2 µl. Each animal received single or multiple injections bilaterally into the visual cortex, and each injection was made over a period of 5–10 minutes. After each injection, the pipette was left in place for an additional 2 minutes before withdrawing it slowly to reduce the possibility that the injected solution would backflow from the site. When the injections were completed, the bone flaps were replaced, the scalp wound was closed with wound clips, the animals were allowed to recover on a warming pad, and then they were returned to their home cages. Three to fifty-six days after the cortical injections, the rats were deeply anesthetized with an intramuscular injection of a mixture of 135 mg/kg of ketamine HCl and 14 mg/kg of xylazine and perfused transcardially with 0.9% NaCl followed by 4% paraformaldehyde in 0.1 M phosphate buffer (PB) at pH 7.4. The brains were postfixed overnight in 4% paraformaldehyde and then sectioned coronally at 75 µm with a vibrating blade microtome (Leica VT 1000S). Sections of this thickness were chosen carefully after experimenting with thicker or thinner sections. In principle thicker sections should allow for the identification of more labeled neurons in each section that appeared to be located entirely within the section. However, sections thicker than 75 µm frequently contained so many labeled neurons that it was difficult to isolate a single labeled neuron in order to reconstruct it. Sections thinner than 75 µm eliminated this problem, but not without raising concern that the dendritic arbors of many labeled neurons had been truncated by the section. It was therefore concluded that 75 µm was the optimum section thickness for the present study.

#### Cytochemistry

Sections of the brain from the level of the optic chiasm through the rostral superior colliculus were collected and washed thoroughly in 0.1 M PB. Transported BDA was detected by an avidin-biotin-peroxidase complex (Elite ABC Kit, Vector Laboratories), and then visualized using 3, 3′-diaminobenzidine tetrahydrochloride (DAB) as a chromogen (Vector Laboratories). Freely-floating sections were washed in 0.1 M PBS, 4 washes of 5 minutes each, and then incubated in PBS containing 0.5% Triton X-100 and 0.1% bovine serum albumin for one hour at room temperature (RT). This was followed by a 30 minute incubation in avidin-biotin-peroxidase complex at RT. After a thorough washing as above, the sections were incubated in a fresh DAB solution (0.005% DAB in 0.1 M PB plus 0.006% H_2_O_2_). In many cases, 0.02% cobalt acetate was added to the DAB solution to intensify the reaction. The sections then were washed four times for 10 minutes each in 0.1 M PB, mounted on chrome alum and gelatin-coated slides, dehydrated, cleared in xylene and coverslipped. Alternatively, some sections reacted with DAB without cobalt acetate toning, were mounted on slides, defatted in xylene, and then rehydrated and processed for gold/silver intensification. The sections were incubated in 1.42% silver nitrate for 1 hour at 56° C, 0.2% gold chloride (HAuCl_4_) for 10 minutes at RT, and fixed in 5% sodium thiosulfate for 5 minutes at RT, with thorough washing between each step. The sections then were dehydrated, cleared in xylene, and coverslipped.

**Figure 2:**
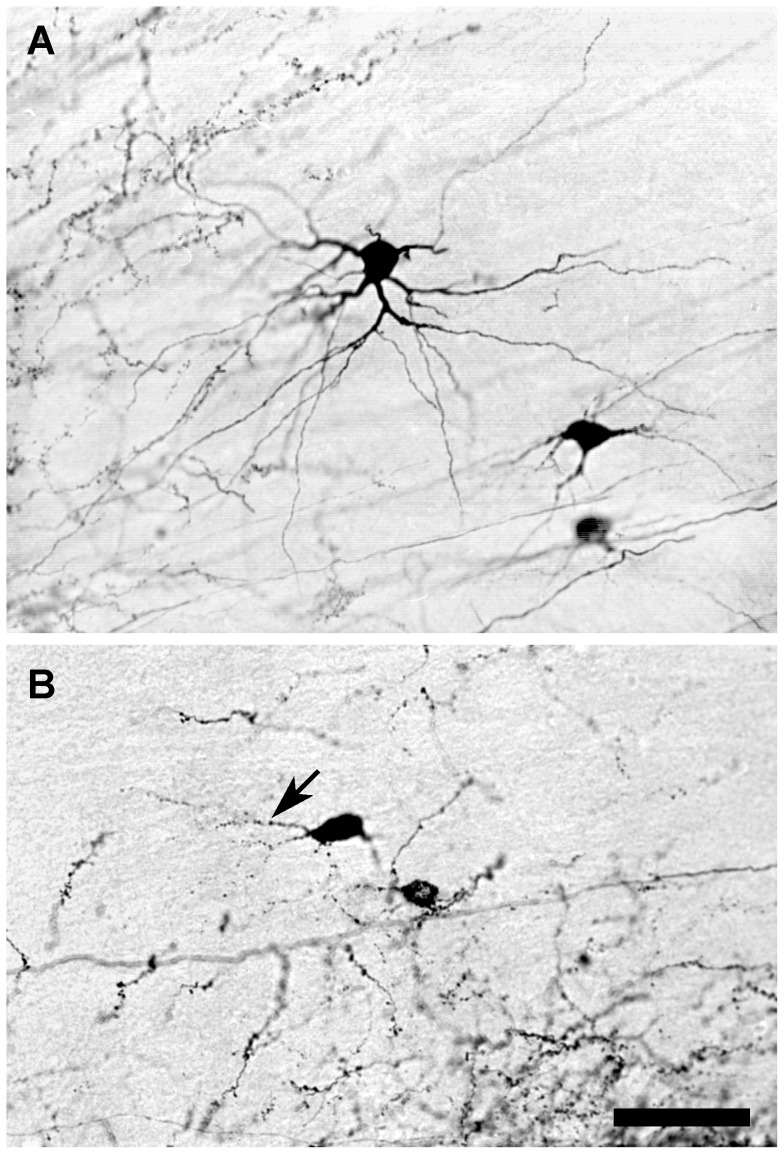
Photomicrographs showing the effects on labeling of two different molecular weights of BDA. (**A**) Fully labeled neurons in the dLGN 3 days after a 0.1 µL injection of 3,000 MW BDA. (**B**) Partially labeled neuronal somas and proximal dendrites (arrow) with granular appearance in the dLGN 3 days after a 0.1 µL injection of 10,000 MW BDA. Scale bar: 60 µm.

The sections were analyzed with a brightfield microscope at different magnifications ranging from 40× to 1000×. Neurons labeled with BDA were drawn at 1000× with the aid of a camera lucida, and specific aspects of their labeling were documented photographically. The quality of BDA-labeled neurons was graded on a subjective scale of 1 to 5. A score of 5 was assigned to neurons that were judged to be fully labeled, as indicated by the complete filling with BDA of the cell soma, as well as the distal processes and fine appendages. At the opposite end of the range, a score of 1 was given to neurons in which the labeling was so sparse that even the cell soma and proximal dendrites were but incompletely filled with BDA. Examples of labeled cells that were scored 5, 3, or 1 can be seen in the following figure panels: 5: Figure 2A, 3: Figure 2B, 1: Figure 4B. The scores for labeled neurons in brains that had been treated similarly were averaged, and the mean scores were ranked to give an estimate of the relative effectiveness of the different treatment conditions that were studied.

## Results

Each BDA injection usually resulted in a cylindrical injection site that varied in diameter from 200 µm to 1.0 mm, depending upon the volume of the injection and the post-injection survival time. In all of the animals examined, most of the neurons in the immediate vicinity of the injection site were labeled heavily with BDA, as were many of the neurons at more distant locations in the cortex and in the thalamus at locations that projected to the injection site. Only those BDA-labeled neurons that lay well outside of the injection site were studied in detail.

### Factors Affecting the Quality of BDA-Labeling

To investigate some of the factors that might influence the quality of neuronal labeling with BDA, we varied several parameters: (a) the molecular weight of the BDA that was injected; (b) the dosage of BDA injected; (c) co-injection of BDA with NMDA, and (d) the length of post-injection survival time. The results of these experiments are summarized in Table 1.

**Table 1:** Effects of three different variables, post-injection survival time, molecular weight, and co-injection with NMDA, on the quality of BDA retrograde neuronal labeling.

Survival Time	Number of Hemispheres	BDA MW	NMDA Concentration	Quality of Labeling
3 days	5	3,000	0	3.9
	2	3,000	10 mM	3.9
	9	3,000	100 mM	4.3
	2	10,000	0	2.8
	2	10,000	100 mM	3.3
7 days	4	3,000	0	4.1
	2	3,000	10 mM	4.2
	2	3,000	100 mM	4.3
	2	10,000	0	3.3
	2	10,000	10 mM	3.2
10 days	4	3,000	0	4.3
14 days	5	3,000	0	3.9
	2	3,000	10 mM	3.3
	2	3,000	100 mM	3.5
	3	10,000	0	2.8
	2	10,000	100 mM	3.0
28 days	2	3,000	0	2.0
	2	3,000	100 mM	1.9
	2	10,000	0	1.6
	2	10,000	100 mM	1.6
56 days	2	3,000	0	1.1
	2	10,000	0	1.0

#### Molecular Weight of BDA Injected

Nineteen animals were injected in one hemisphere with 3,000 MW BDA and with 10,000 MW BDA in the other hemisphere. The rats were allowed to survive for 3, 7, 14, 28, and 56 days before being perfused as described above and their brains processed for BDA labeling of neurons. Great care was taken to ensure that the BDA injections in each hemisphere were matched as closely as possible, and that the processing of all sections was identical so that any consistent variation in neuronal labeling could be attributed to the molecular weight of the BDA that had been injected.

In each of the animals studied, BDA-labeled cells and axons were found bilaterally in the cortex and in the thalamus. In 5 of 19 cases, injections with 10,000 MW BDA resulted in larger apparent injection sites in the cortex and denser neuronal labeling in the thalamus than was seen on the contralateral side of the brain in which 3,000 MW BDA had been injected. However, the majority of labeled cortical and thalamic neurons were not filled solidly after injection with 10,000 MW BDA. Instead, only the cell somas and proximal dendrites of many retrogradely labeled neurons were filled, and the labeling often was granular in appearance. In contrast, labeling with 3,000 MW BDA generally yielded more fully labeled neurons and axon terminals, particularly in the thalamus, but the difference in the quality of neuronal labeling with 3,000 and 10,000 MW BDA was only apparent in animals that survived for 14 days or less.

In animals that survived for 3 days after cortical injections of BDA, fully labeled neurons were observed in both cortices, but more labeled neurons were seen in the hemisphere in which 3,000 MW BDA had been injected than in the hemisphere that had received an injection of 10,000 MW BDA. In the thalamus, fully labeled neurons were seen at 3 days survival only ipsilateral to the cortex that had received an injection of 3,000 MW BDA (Fig. 2A). By contrast, in the thalamus ipsilateral to the cortex that had received an injection of 10,000 MW BDA, labeled neurons were not filled in their distal processes, and the labeling in proximal dendrites frequently was granular (arrow in Fig. 2B). Following cortical injections with 3,000 MW BDA, anterogradely labeled axons in the thalamus were filled solidly and their terminal arbors appeared to be delineated completely. However, when 10,000 MW BDA was injected into the cortex, anterogradely labeled axons were not filled solidly, but instead contained granules of BDA.

Similar results were observed in rats that survived for 7–14 days. Injections with 3,000 MW BDA yielded many fully labeled neurons in both the cortex and the thalamus, while 10,000 MW BDA injections produced fully labeled neurons only in the cortex. Neurons in the thalamus labeled with 10,000 MW BDA often were incompletely filled. After survival times of 28 or 56 days, the observed labeling of cortical and thalamic neurons with either 3,000 or 10,000 MW BDA was light and granular, with no marked difference in labeling quality that could be attributed to a difference in the molecular weight of the BDA injected (Table 1).

#### Injection Dosage

**Figure 3:**
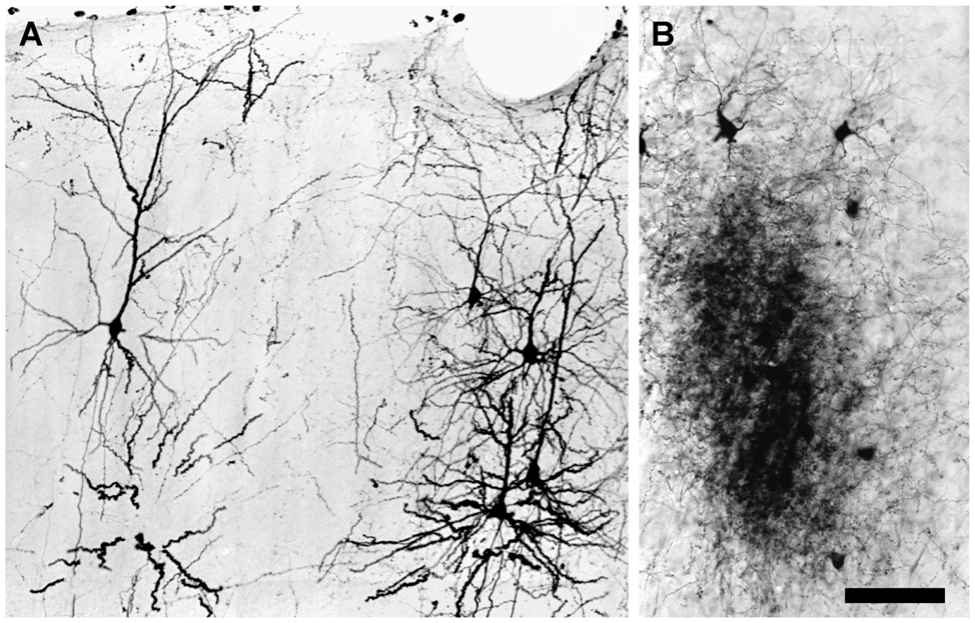
Photomicrographs showing differences in the density of BDA labeling in the cortex and dLGN 3 days after a 0.4 µL injection of 3,000 MW BDA into the visual cortex. (**A**) Scattered BDA-labeled neurons in the visual cortex. Individual neurons can be distinguished easily from their neighbors. (**B**) A dense patch of BDA labeling in the dLGN. The patch comprises corticothalamic axons and dLGN neurons and processes. The overlap of labeled neurites makes it impossible to distinguish individual details. Scale bar: 75 µm in **A**, 60 µm in **B**.

To investigate the relation between injection dosage and the quality of neuronal labeling, the volume of single injections of 3,000 MW BDA was varied from 0.02 to 0.4 µl. Large injections of 3,000 MW BDA, 0.2–0.4 µl, always produced more neuronal labeling than small ones. In the visual cortex, BDA labeling after a large injection was widespread, and extended into neighboring cortical areas, such as the parietal and temporal cortices, where many fully labeled neurons could be seen (Fig. 3A). In the thalamus, BDA-labeled neurons usually appeared in clusters, particularly in the dLGN. Within large dense clusters of labeled neurons (Fig. 3B), retrogradely labeled cells and anterogradely labeled axon arbors were intermingled, and it was impossible to visualize single fully labeled neurons in isolation.

By contrast, small cortical injections of 3,000 MW BDA, 0.02 µl, reduced the size and density of labeled patches in the thalamus, and also yielded fewer labeled neuronal cell somas, and rarely were distal dendrites of labeled neurons filled. Even when up to 13 small injections involving the full extent of area 17 of the visual cortex were made in a single hemisphere, no fully labeled thalamic neurons ever were observed. Taken together, these results indicate that the labeling of thalamic neurons with a cortical injection of BDA was optimum with an injection volume of 0.1 µl of 3,000 MW BDA. An injection of this volume produced moderately dense patches of labeled cells with isolated neurons that were fully labeled.

#### Co-injection of BDA with NMDA

It has been reported that co-injecting NMDA with BDA can increase the number of neurons that are retrogradely labeled, perhaps by stimulating the uptake of BDA by axon terminals [48]. To explore this possibility further, a series of rats was injected with 3,000 MW BDA alone in one hemisphere and with a mixture of BDA and NMDA (10 or 100 mM) in the other hemisphere. When NMDA was used at a concentration of 100 mM, 1∶1 v/v with BDA, the number of retrogradely labeled neurons increased in both the cortex and thalamus, compared with the number seen when BDA was injected by itself. The greatest difference in labeling was seen in rats that survived for 3 days after the cortical injections. In these animals, more fully labeled neurons were seen in the thalamus with combined NMDA/BDA injections than with BDA injections alone. However, this difference in the frequency of labeling diminished with longer survival times. In animals that survived for 14 days or more after an injection, no clear difference in the efficiency of labeling with a combined NMDA/BDA injection was observed.

With a low concentration of NMDA, 10 mM, there was no consistent difference at any survival time between combined NMDA/BDA injections and single BDA injections. Of the 8 rats that were studied, 4 displayed labeling that favored combined NMDA/BDA injections, 3 showed no difference, and one gave results with BDA alone that were superior to BDA combined with NMDA. On balance, combining NMDA with BDA may result in labeling that is superior to that seen with BDA alone. However, in our hands an improvement in labeling when combining NMDA with BDA was not observed reliably when a low concentration, 10 mM, of NMDA was used. Although improved labeling was seen when a higher concentration, 100 mM of NMDA was combined with BDA, the improvement was evident only in rats that survived for 3 days after a combined BDA/NMDA injection.

#### Post-injection Survival Time

The quality of BDA labeling was related closely to post-injection survival time (Table 1). In rats that survived for 3–10 days after cortical injections with 3,000 MW BDA, fully labeled neurons were seen in the cortex and thalamus. At 14 days survival, the number of fully labeled neurons observed in the thalamus had declined and many labeled cells had distal dendrites that were incompletely filled. At 28 days after injection, some cortical neurons located near the injection site were fully labeled, but the cell somas and proximal dendrites of thalamic neurons were only partially labeled, and no thalamic neurons displayed labeling of their distal dendrites. Fifty-six days after an injection of BDA, only a few partially labeled cell somas with short proximal dendrites were observed in the cortex and the thalamus.

**Figure 4:**
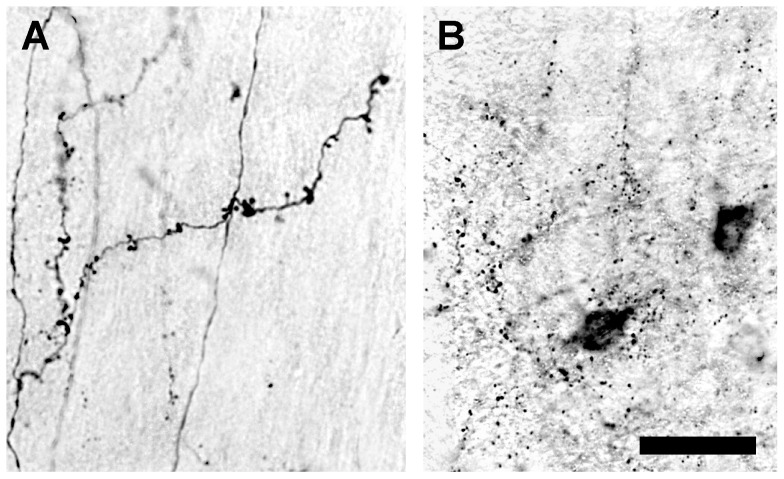
Photomicrographs showing the effects of varying post-injection survival time on the quality of BDA labeling. (**A**) Fully labeled corticogeniculate axon arbors 28 days after a 0.1 µL injection of 3,000 MW BDA. (**B**) At 56 days after injection, BDA labeling is punctate and largely cleared from labeled processes. Scale bar: 40 µm.

Anterogradely labeled axons and their terminals were seen in the cortex and thalamus 3 days after a 3,000 MW BDA injection, but generally only large caliber axons and their arbors were fully labeled. Fine axons and their arbors and terminal boutons were labeled with a granular appearance. At 10 days survival however, many fine axons were solidly filled with BDA, and displayed fine morphological detail. Fully labeled axons could be seen at 28 days post-injection (Fig. 4A), but at 56 days only lightly labeled axons presenting a granular appearance (Fig. 4B) were evident.

### Detailed Morphology of BDA-Labeled Neurons and Axons

#### Neocortex

**Figure 5:**
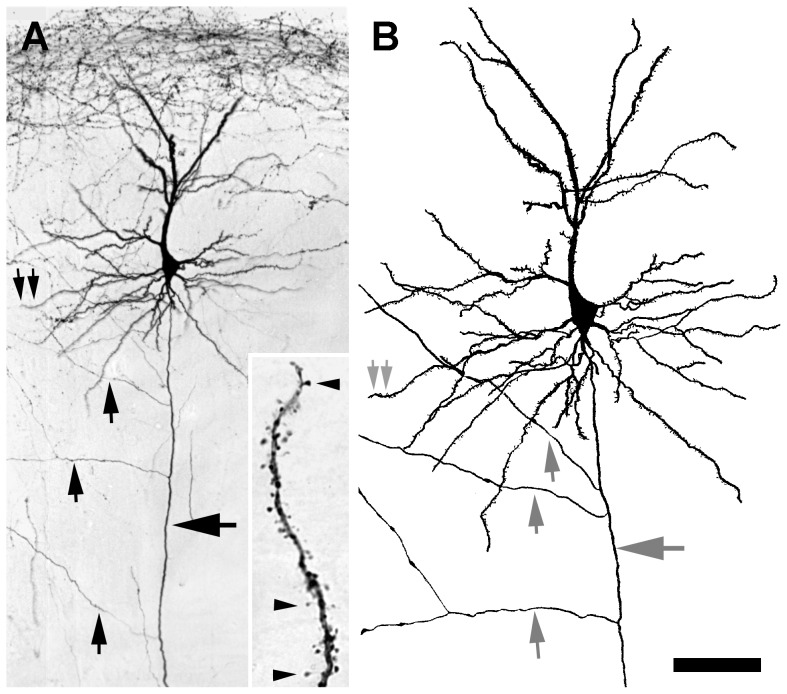
Pyramidal neuron in layer II of the cortex 3 days after a 0.1 µL injection of 3,000 MW BDA. (**A**) The axon of the pyramidal neuron (horizontal arrow) descends to the white matter, but also sends branches (vertical arrows) horizontally. Double arrows indicate the region shown in the inset, rotated 90° clockwise, that illustrates spiny appendages (arrowheads) distributed along a segment of a labeled dendrite. (**B**) Camera lucida drawing of the labeled neuron shown in **A** to illustrate the entire dendritic arbor of the neuron. Scale bar: 75 µm in **A**, 50 µm in **B**, and 10 µm for the inset in **A**.

More than 90% of the cells labeled in the cortex were pyramidal cells with prominent apical dendrites that often could be traced up to 1200 µm from the cell soma before they ramified radially up to 300 µm. The primary basal dendrites of pyramidal cells usually branched in the vicinity of the cell body, and the higher order dendritic branches could be followed for up to 300 µm. Labeled axons could be identified by their smooth surface and relative homogeneous caliber (large arrow in Fig. 5A, B). Labeled non-pyramidal neurons also were observed, largely in upper layer II or layer I of the cortex. The dendrites of pyramidal and non-pyramidal neurons often were studded with spines (Fig. 5A, inset). Most of the labeled spines consisted of a short stalk of about 2 µm in length that terminated in an ovoid head about 1 µm in diameter. No consistent differences were noted in the shapes or density of the spines between pyramidal and non-pyramidal cells, although they varied in detail within each group of cells.

#### Thalamus

**Figure 6:**
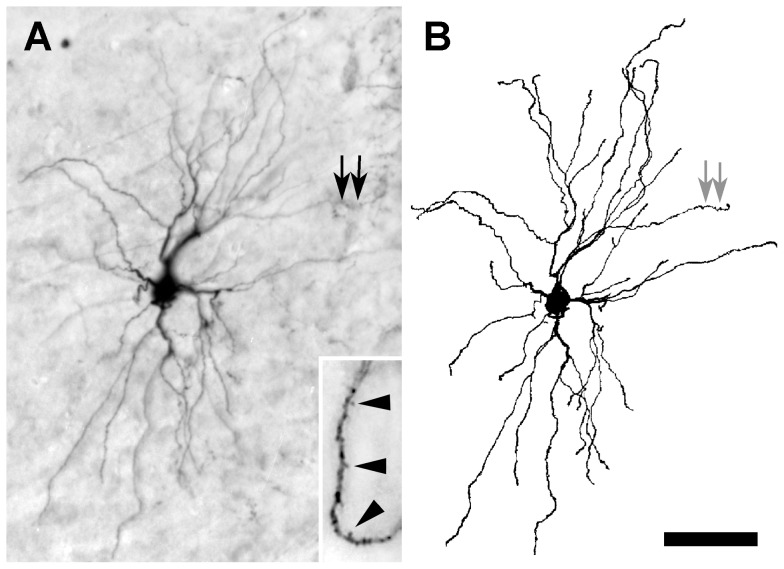
Projection neuron in the dLGN 3 days after a 0.1 µL injection of 3,000 MW BDA. (**A**) Inset shows at higher magnification a segment, rotated 90° clockwise, of the dendrite indicated by arrows in **A** and **B** illustrating tuft-like appendages (arrowheads). (**B**) Camera lucida drawing of the labeled neuron shown in **A** to clarify the full extent of the dendritic arbor. Scale bar: 50 µm in **A** and **B**, and 10 µm for the inset in **A**.

Fully labeled neurons were observed mainly in the dorsolateral region of the thalamus, especially in the dLGN. A typical, fully labeled thalamic neuron had a cell soma with a cross-sectional area of about 200 µm^2^, from which 2–6 primary dendrites emerged (Fig. 6A, B). The primary dendrites often branched in the vicinity of the soma at acute angles, each giving rise to 2–4 slender secondary dendrites that extended up to 300 µm. Over 70% of BDA-labeled neurons did not have primary dendrites that emerged from various sites around the entire perimeter of the cell soma, regardless of the cell's location in the thalamus. The most common type of dendritic appendages seen on thalamic neurons were tuft-like protuberances of uniform shape and largely distributed along the distal portions of secondary or higher order dendrites (Fig. 6A, inset).

#### Cortico-cortical and Corticothalamic Axons

**Figure 7:**
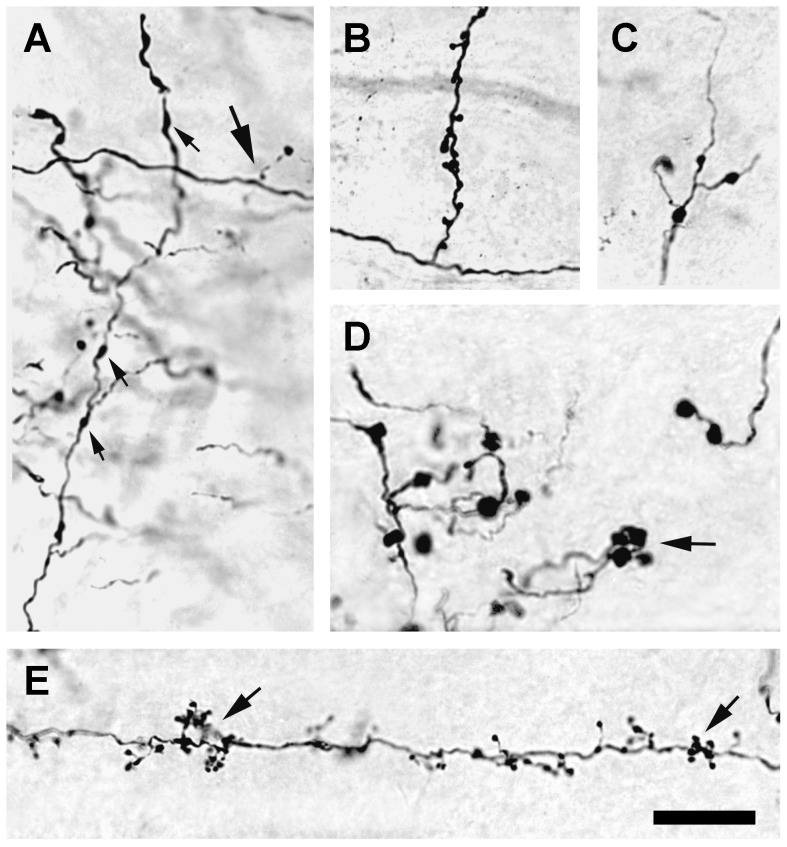
Micrographs of axons 3 days after a 0.1 µL injection of 3,000 MW BDA. (**A**) Corticocortical axons with varicosities (upward arrows). Occasionally, short branches that terminate in round spherules (large arrow) also were seen. (**B**) Corticogeniculate axon with an atypically long side branch studded with boutons. (**C**) Corticothalamic axon in the lateral dorsal nucleus, with small diameter varicosities (2–3 µm) and short appendages. (**D**) Corticothalamic axons in the lateral posterior nucleus of the thalamus with many large varicosities (>4 µm in diameter) and boutons that often were seen in clusters (arrow). (**E**) Typical fine caliber, corticogeniculate axon is beaded with small varicosities, and supports many dense, short appendages that end in boutons (arrows) that frequently are arrayed in clusters. Scale bar: 20 µm.

Anterogradely labeled axons could be followed from the site of BDA injection in the visual cortex to other cortical areas and to the thalamus. Labeled cortico-cortical axons usually had fine collaterals that were studded with ovoid varicosities (Fig. 7A). The shape, size, and density of axon terminal swellings appeared to vary within each cortical region. Labeled corticothalamic axons varied in their morphology. The majority of corticothalamic axons displayed fine collaterals on which numerous short stalks, each ending in a spherical bulb, could be seen (Fig. 7E). The stalks were distributed unevenly and frequently appeared grouped in clusters (arrow in Fig. 7E). Occasionally, collateral branches extended directly from a corticothalamic axon, such as those observed in the dLGN, and each collateral branch often supported large appendages (Fig. 7B). In the lateral dorsal nucleus of the thalamus, labeled axons were seen with fine collaterals that were beaded with small varicosities of about 2.5 µm in diameter, but lacked appendages (Fig. 7C). Axons with thick collaterals, studded with large varicosities that were greater than 4 µm in diameter, were found predominantly in the lateral posterior nucleus (Fig. 7D).

## Discussion

BDA has been used extracellularly in the CNS for many years as an anterograde and retrograde tracer. Generally, it is thought that 10,000 MW BDA is suitable primarily for anterograde tracing, while 3,000 MW BDA is preferred for retrograde labeling [1], [26], [29]. However, we are unaware of any prior study that compared 10,000 MW BDA with 3,000 MW BDA to determine if this consensus regarding the applicability of high and low molecular weight BDA is correct. Moreover, we were interested to learn if typical BDA labeling could be enhanced to reveal the detailed structure of axons and neuronal cell somas and dendrites, because an extracellular method with this capability would be very useful. Therefore, we varied several parameters associated with the extracellular administration of BDA and the subsequent resolving of BDA-labeled axons and neurons, and evaluated systematically whether BDA could be optimized to fill axons and neurons in the CNS fully; specifically, to show the fine details of their morphology with the same resolution that can be obtained with Golgi staining or by the injection of an intracellular label. These experiments have led us to conclude that 3,000 MW BDA is superior to 10,000 MW BDA for anterograde and retrograde tracing as well as revealing fine structural detail of labeled axons and neurons.

### 

#### Volume of BDA Injected

At the concentration of BDA used in this study, 5% w/v, a cortical injection of BDA smaller than 0.05 µl was not sufficient to label fine neuronal profiles. The reason for this deficiency is not known, but it is possible that very small injections create insufficient damage at the injection site to promote the uptake of injected BDA. Alternatively, very small injections may deliver too little BDA to fill labeled profiles completely, or they are filled but with insufficient density to be visualized with the cytochemical techniques used in the present experiments.

A large injection of BDA may overcome these problems, but if the injection volume is too large, then the density of anterograde and retrograde labeling can be so intense that labeled neurons and axons appear in congested clusters, and individual details cannot be resolved. For example, a large BDA injection in visual cortex of 0.2–0.4 µl yields many fully labeled neurons in the dLGN, but these labeled neurons overlap to such an extent that their individual features are difficult or impossible to analyze.

On balance, the best retrograde labeling of neurons with BDA was obtained with cortical injection volumes of 0.1 µl. Injections of this volume labeled individual neurons in the thalamus fully, but the density of labeled neurons was low enough that single labeled cells could be studied with little or no overlapping of their dendrites by neighboring labeled cells.

#### Molecular Weight of BDA Injected

Although BDA has been available for many years in different molecular weights, previous labeling studies generally have used BDA with an unspecified molecular weight or a molecular weight of 10,000 [1], [20]–[29] although others have reported that low molecular weight BDA diffuses faster than high molecular weight BDA, and also fills fine profiles better [49], [50]. One possible reason for choosing high molecular weight BDA as a labeling agent is that it may diffuse more slowly than low molecular weight BDA and, therefore, may persist longer inside of a labeled cell. However, we are not aware that this hypothesis ever has been tested directly nor has the labeling capability of low molecular weight BDA been compared systematically with that of high molecular weight BDA in controlled experiments prior to the current report.

Our experiments confirm that when all other conditions are equal, 3,000 MW BDA is superior in all respects to 10,000 MW BDA for completely labeling axons and neurons in the CNS. Moreover, we do not have any evidence that high molecular weight BDA persists longer of BDA, poor quality labeling was observed with either 3,000 MW or 10,000 MW BDA.

#### Post-Injection Survival Time

Intracellularly transported BDA is thought to be cleared slowly, and has been considered a suitable long-term label for living cells. In previous studies using high molecular weight BDA, anterogradely labeled axons were seen for up to 23 days after injection in rats, and up to 7 weeks in monkeys [20]. Retrogradely labeled neurons have been reported to persist for up to two weeks without significant loss of detail [22].

In the present study, we also noted a difference in the persistence of BDA in labeled axons and neurons. For example, three days after injection of BDA, fully labeled neurons can be visualized clearly, but at this time, only large diameter axons are fully labeled in most animals. Two weeks after injection of BDA, many labeled neurons had begun to lose detail in their distal dendrites, but completely labeled axons were evident for up to four weeks. Thus, the clearing of BDA appears to be underway after 14 days in retrogradely labeled neurons and after 28 days in anterogradely labeled axons. Labeled axons and cells first lose fine detail, and then they acquire a granular appearance before most of the label is lost entirely. Collectively, these results suggest that the anterograde labeling of axons may proceed at a slower rate than the retrograde labeling of neurons. Similarly, the BDA accumulated in axons appears to be cleared more slowly than that in retrogradely labeled cell somas and dendrites, indicating that the optimum post-injection survival time will depend on whether the purpose of using BDA is to label axons that leave the injection site or neurons that project to it.

#### Co-injection of BDA with NMDA

A number of reports have shown that the retrograde labeling of neurons with horseradish peroxidase and wheat germ agglutin-HRP is activity dependent. Increased neuronal activity at the injection site appears to enhance the uptake of HRP by local neurons or by neurons that project to the site [51]–[56]. In the rat visual cortex, glutamate is used as a neurotransmitter by many neurons, and NMDA receptors are abundant throughout this region [56]–[60]. In a prior report, 10–100 mM NMDA was co-injected with biocytin or BDA into the visual cortex to raise the level of neuronal activity at the injection site [48]. This increase in activity improved the retrograde labeling of nearby cortical neurons by nearly 4 fold, independent of the concentration of NMDA that was used.

In agreement with these earlier results, we also found that co-injection of BDA with 100 mM NMDA produced more retrogradely labeled neurons than did the injection of BDA alone. However, the increase in the number of labeled neurons that we saw when NMDA was co-injected with BDA was less than previously reported, and was observed reliably only in rats that survived for short periods of time after the injection. As survival time increased, the effect of co-injecting NMDA with BDA diminished. When low concentrations of NMDA, 10 mM, were co-injected, only a modest improvement in neuronal labeling was detected in slightly more than half of the animals, 5 of 8, that were studied.

Moreover, significant NMDA-induced cell death at and near the BDA/NMDA injection site and in its vicinity has been reported [48]. In view of this, one must be concerned that the morphology of neurons projecting to the injection site will be altered by damage to their target. Thus, our experiments suggest that NMDA may enhance BDA labeling, but only when NMDA is used at high concentrations, which may trigger excitotoxic cell death, and in studies that employ relatively short post-injection survival times, at least 3 days but not more than 14 days.

#### Capability of BDA Labeling to Reveal the Complete Morphology of dLGN Projection Neurons

Although a cortical injection of BDA has been demonstrated to label many local cortical neurons fully [48], the capability of BDA to label neurons in detail that are located distant to a cortical injection site, e.g. in the thalamus, has not been demonstrated previously to the best of our knowledge. In the present study, we have confirmed earlier work that BDA can be used to reveal the fine morphological details of neurons in the vicinity of an injection site, and, in addition we have shown that BDA, when injected following an optimal protocol, also is suitable for labeling many thalamic neurons in detail that project to the injection site.

In the cortex, we found that BDA retrogradely labels more pyramidal than non-pyramidal neurons. This result is consistent with previous reports showing that pyramidal cells are the major contributors to cortico-cortical connections [45], [61], [62]. The present findings also confirm reports demonstrating that some non-pyramidal cells make cortico-cortical connections [63], [64].

The morphology of dLGN neurons has been examined in Golgi-impregnated material and in HRP and biocytin-filled cells [30]–[35]. Our retrograde labeling results with BDA generally agree with previous reports concerning the morphology of dLGN neurons and, in addition, have allowed us to define for the first time the dLGN neurons that are thalamocortical projection neurons, and to provide new information about the sizes and spatial organization of their dendritic arbors. For example, the dendrites of some dLGN projection neurons span up to 300 µm [65], which is 50% greater than has been reported previously in the rat or mouse [31], [66].

One of the salient features of BDA, when used as described here, is its ability to reveal fine details of neuronal structure. Differences between cortical and thalamic neurons in the morphology of dendritic appendages can be resolved with a clarity normally associated with intracellularly labeled neurons. The characteristic spines of cortical dendrites can be distinguished readily in shape and size from the predominantly short, tufted stalks that protrude from the distal dendrites of thalamic neurons. Axon arbors labeled with BDA in the present study display sufficient fine detail that distinctions can be made among axons of different types. Morphological differences in corticothalamic axons from the primary visual cortex in the rat have been used to classify these axons into two types [46]. In one type of axon, fine collaterals are seen that support delicate appendages that often end in boutons (Fig. 7B, C). Other axons contain small varicosities with fine collaterals that end in boutons (Fig. 7E). A third type of corticothalamic axon, which has been documented previously in the hamster [67], but not in the rat, is revealed by BDA labeling to have large varicosities from which medium caliber collaterals and boutons originate (Fig. 7D).

In summary, the present results demonstrate that BDA can be injected extracellularly and used effectively as a label to resolve very fine details of neuronal morphology. When attention is paid to the volume and the molecular weight of BDA that is injected, and to the post-injection survival time of the animal, BDA staining, intensified with gold and silver or cobalt acetate, is capable of labeling axons and neurons in the intact brain with a high degree of fidelity, and with a clarity that normally is associated with intracellular labeling methods.
